# FLex: joint pose and dynamic radiance fields optimization for stereo endoscopic videos

**DOI:** 10.1007/s11548-025-03446-6

**Published:** 2025-07-21

**Authors:** Florian Stilz, Mert Karaoglu, Felix Tristram, Nassir Navab, Benjamin Busam, Alexander Ladikos

**Affiliations:** 1 Chair for Computer Aided Medical Procedures, Technical University of Munich, Boltzmannstr. 3, 85748 Garching, Germany; 2 Imfusion GmbH, Agnes-Pockels-Bogen 1, 80992 München, Germany

**Keywords:** 3D Reconstruction, Neural rendering, Robotic surgery, Camera pose optimization

## Abstract

**Purpose:**

Reconstruction of endoscopic scenes is crucial for various medical applications, from post-surgery analysis to educational training. However, existing methods are limited by static endoscopes, restricted deformation, or dependence on external tracking devices for camera pose information.

**Methods:**

We present flow-optimized local hexplanes (FLex), a novel approach addressing the challenges of a moving stereo endoscope in a highly dynamic environment. FLex implicitly separates the scene into multiple overlapping 4D neural radiance fields (NeRFs) and employs a progressive optimization scheme for joint reconstruction and camera pose estimation from scratch.

**Results:**

Tested on sequences of length up to 5000 frames, which is five times the length handled in the experiments of previous methods, this technique enhances usability substantially. It scales highly detailed reconstruction capabilities to significantly longer surgical videos, all without requiring external tracking information.

**Conclusion:**

Our proposed approach overcomes key limitations of existing methods by enabling accurate reconstruction and camera pose estimation for moving stereo endoscopes in challenging surgical scenes. FLex’s advancements enhance the applicability of neural rendering techniques for medical applications, paving the way for improved surgical scene understanding. Code and data will be released on the project page: https://flexendo.github.io/

**Supplementary Information:**

The online version contains supplementary material available at 10.1007/s11548-025-03446-6.

## Introduction

Visually and geometrically accurate reconstructions of surgical scenes are crucial for various computer vision and AR/VR applications such as post-surgical longitudinal assessment [1], surgical training [2], and data generation for other learning-based computer vision and robotics applications [3]. However, endoscopic videos present a range of visual and practical challenges, including strong non-homeomorphic deformations, prolonged recording times, and the difficulty of determining camera positions. These challenges often lead to a reliance on external tools for acquisition, diminishing the ease of use and practicality of the reconstruction frameworks.

Prior methods widely explore usage of explicit representations such as sparse and dense point clouds [4, 5] in visual odometry and simultaneous localization and mapping (SLAM) frameworks. Even though these approaches often provide efficient solutions for combined camera tracking and reconstruction, their incomplete geometry modeling results in limitations when rendering views from new camera poses. Our method is similar regarding joint optimization of localization and reconstruction; however, we instead use a dynamic neural radiance fields (NeRF) [6]-based architecture to reconstruct the scene together with capturing the dynamics, thereby enabling high-quality time-dependent novel view synthesis.

Following the line of EndoNerf [7], various NeRF [8–11] and Gaussian splatting (GS)-based [12–15] methods, developed in parallel to our work, extensively study adaption of neural representations for dynamic endoscopic scenes. While these works present great results and a promising research direction, unlike our method, they rely on external, reliable measurement or computation of the camera poses which are difficult to obtain in endoscopic environments.

The challenge in obtaining reliable camera poses in endoscopic environments lies in the strong deformations of the entire scene, which introduce ambiguities in camera pose estimation. While there are some interesting recent  [16–18] and concurrent works [19, 20] that show great promise in tackling the joint optimization of camera pose and neural representation in both non-medical and endoscopic scenes, respectively, despite their important contributions, they all assume a static scene without deformations. In our experiments on highly deformable laparoscopic scenarios we demonstrate that using neural representations which cannot model scene deformations leads to a decline in both novel view synthesis quality and camera pose accuracy.

To solve this issue, we present FLex, a novel NeRF-based architecture capable of high-quality novel view synthesis from pose-free surgical videos, containing strong deformations. Employing a progressive optimization scheme [18], FLex utilizes local dynamic radiance fields to jointly optimize for pose and scene representation. Our approach improves usability by eliminating the need for external tracking devices and scales high reconstruction capabilities to handle in theory boundlessly long surgical videos as demonstrated in our experiments on sequences of up to 5000 frames, which is five times the length handled in the experiments of the concurrent work [13]. In the context of neural reconstruction applications on dynamic endoscopic scenes, along with a contemporary work following a different approach, BASED [21], we believe FLex is the first to investigate joint pose optimization. In addition to architectural differences, in this work we jointly target efficient scaling to longer sequences.

In evaluating FLex, we perform experiments on surgical recordings featuring a moving camera with no prior pose information, along with tissue deformations. Extensive tests on the StereoMIS [22] dataset demonstrate that FLex significantly improves the quality of novel view synthesis while maintaining competitive pose accuracy, showcasing its potential for practical surgical applications.

To summarize, our contributions are:

- A novel NeRF architecture for 4D reconstruction in highly dynamic endoscopic scenes without the need for camera pose information, achieved through optical flow supervision and joint optimization in a progressive strategy.
- Extending existing scene scaling methods to 4D, enabling high reconstruction quality of in theory boundlessly long *dynamic* surgical videos.
- State-of-the-art performance in novel view synthesis, while being the first method to jointly optimize camera poses in dynamic environments, achieving pose accuracy competitive with dedicated pose estimation frameworks on the challenging StereoMIS dataset.

**Fig. 1:**
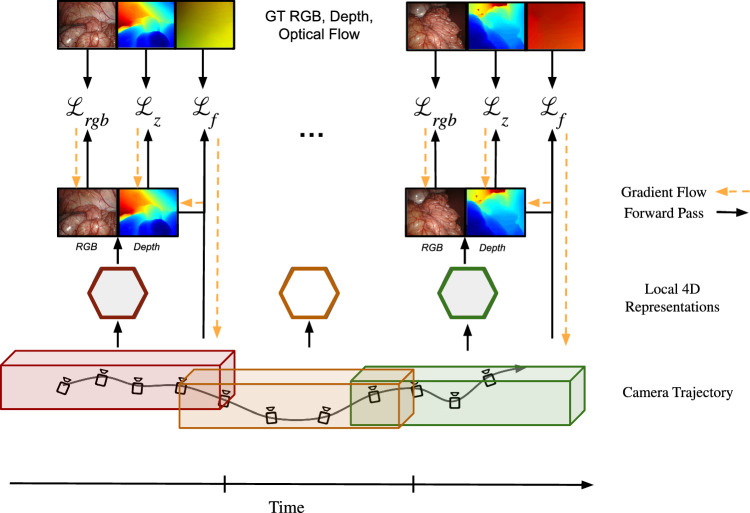
Joint progressive pose and local dynamic radiance fields optimization. Spatial extents clustered within the bounding boxes of different colors represent the spatio-temporal domain of the corresponding local radiance fields. We obtain depth images and forward/backward optical flow from stereo images using RAFT [23] and use it during our optimization. For a given camera pose we render color and depth images from our local dynamic models and supervise them with the losses outlined in Sec. 2.4. The predicted depth image is also used for the optical flow loss as described in Eq. (7). Yellow arrows symbolize gradient flow used for optimization

## Method

### Overview

Given a rectified stereo endoscopic video, our goal is to reconstruct the 4D scene accurately without prior camera pose information. For this, we propose a new method **FLex**, standing for **F**low-optimized **L**ocal H**ex**planes, depicted in Fig. 1, which combines advancements from recent NeRF literature to build multiple smaller dynamic models that are progressively optimized. In contrast to prior work [7, 8, 10], we do not have one unified representation of the scene but multiple smaller overlapping ones. The representation of local models allows us to represent boundlessly large scenes, both temporally and spatially, accurately without incurring prohibitive memory growth while maintaining a high feature grid resolution. Furthermore, adopting a progressive optimization scheme enables the optimization of poses from scratch. Since endoscopic environments often have textureless surfaces which make geometry optimization from photometric consistency difficult we additionally incorporate supervision through optical flow and stereo depth priors.

### 4D scene representation

NeRFs [6] implicitly model a 3D scene utilizing differentiable volume rendering to predict pixel colors. They can be adapted to a 4D scene representation by adding the timestep *k* as an additional input to the model. The color $\hat{C}(\textbf{r})$ of a ray $\textbf{r}=\textbf{o}+\textbf{d}t$ with origin $\textbf{o}$ and direction $\textbf{d}$ at timestep *k* is calculated from the density $\sigma$ and color *c* that are output from the model with volume rendering:

1 $$\begin{aligned} \hat{C}(\textbf{r}) = \int _{t_n}^{t_f} w(t) \cdot c(t) \textbf{d}t, w(t) = \exp \left( -\int _{t_n}^{t} \sigma (s) ds \right) \cdot \sigma (t). \end{aligned}$$

Using these rendering weights *w*, the expected termination depth $\hat{z}$ of the ray $\textbf{r}$ can be calculated by taking the weighted sum of each sampling location *t* along the ray between $[t_{n},t_{f}]$.

2 $$\begin{aligned} \hat{z} = \int _{t_n}^{t_f} w(t) \cdot t\ \textbf{d}t \end{aligned}$$

The continuous integral in Eqs. (1), (2) is discretized following [6]. We choose HexPlane [24] as our local model, which represents a dynamic scene using an explicit 4D feature grid paired with an implicit MLP. The grid is constructed from several planes, where each plane represents a combination of two dimensions, three spatial dimensions and one temporal dimension, yielding six planes in total. During ray-casting, the corresponding features on each plane are extracted for the spatio-temporal sampling locations along the ray and combined by multiplication and concatenation before being fed into smaller MLPs and similarly to NeRF [6] rendered with volumetric rendering. For more details regarding HexPlanes [24] architecture we refer the reader to the original publication.

### Progressive optimization

Endoscopic videos contain two main challenges for NeRF architectures: They are dependent on external tools for accurate pose estimation and can constitute arbitrarily long sequences of a dynamic environment. In order to tackle these two problems in a robust and efficient way, our joint pose and radiance fields optimization scheme *combines* the concepts of *progressive optimization* and dynamic allocation of *local HexPlane models* as visualized in Fig. 1 and inspired by LocalRF [18].

In the scope of progressive optimization, we start with the first five frames of the sequence, in which the first frame of the video is assigned an identity pose matrix, then we consecutively add one frame at a time, initializing its camera pose parameters with the previous frame’s camera pose. When the appended frame increases the number of frames above a preset threshold, $t_{k}$, or the distance between the optimized position of the camera and the center of the current local model is larger than a distance threshold, $t_d$, we instantiate a new local model, setting this new frame to be its origin. To ensure consistency across local models, we assign the last thirty frames of the previous model to be overlapping with the new local model. To secure a coherent trajectory during progressive optimization, we consistently sample rays from the last four appended frames. During inference, if a pose corresponds to the spatial and temporal extent of multiple local models, each model’s contribution is aggregated into the ray-casting formulation with blending weights linearly set on the overlapping regions based on the proximity to the centers of the local models. Before a new local model is initialized, the last model goes into a refinement phase where the pose and model parameters are optimized with batches of samples uniformly picked along its entire span.

### Training objectives

We employ the common photometric loss $\mathcal {L}_{rgb}$ as defined in Eq. (3) with ground-truth $C(\textbf{r})$ and predicted $\hat{C}(\textbf{r})$ pixel values for ray $\textbf{r}$ within the set of rays $\mathcal {R}$:

3 $$\begin{aligned} \mathcal {L}_{rgb} = \frac{1}{|\mathcal {R}|}\sum _{\textbf{r}\in \mathcal {R}}\left\| \hat{C}(\textbf{r}) - C(\textbf{r})\right\| _{2}^{2} \end{aligned}$$

We also employ geometric regularizers by applying a depth supervision loss along with line-of-sight priors as introduced by Rematas et al. [25]. We denote them together as $\mathcal {L}_{z}$, composed as $\mathcal {L}_{z} := \mathcal {L}_\textrm{depth} + \mathcal {L}_\textrm{near} + \mathcal {L}_\textrm{empty}$.

For the depth loss, the predicted depth $\hat{z}$ for ray $\textbf{r}$ is optimized with the ground-truth depth *z* via an L2 loss:

4 $$\begin{aligned} \mathcal {L}_\textrm{depth} = \frac{1}{|\mathcal {R}|}\sum _{\textbf{r}\in \mathcal {R}}\Vert \hat{z}(\textbf{r})-z(\textbf{r})\Vert _{2}^{2} \end{aligned}$$

The line-of-sight priors regularize the density values along a ray to be concentrated on the actual surface, thereby, together with the depth loss, improving the capture of the scene geometry. Given a set of data samples *D*, $\mathcal {L}_\textrm{near}$ ensures that the rendering weights *w*(*t*) around the region of *z* follow a Gaussian distribution, where $\mathcal {K}_{\epsilon }(x) = \mathcal {N}(0, (\epsilon /3)^2)$ (see Eq. (5)). $\epsilon$ is initially set to be 1 cm and, over time, exponentially reduced to 1 mm.

5 $$\begin{aligned} \mathcal {L}_\textrm{near} = \mathbb {E}_{\textbf{r} \sim \mathcal {D}} \left[ \int _{z(\textbf{r})-\epsilon }^{z(\textbf{r})+\epsilon } \left( w(t) - \mathcal {K}_{\epsilon }(t-z(\textbf{r}))\right) ^2 dt \right] \end{aligned}$$

Additionally, a penalty is added for the rendering weights *w*(*t*) in the empty region to push them toward zero, as depicted in Eq. (6). The empty region is defined from the nearest sampling point $t_{n}$ until the surface region is covered in Eq. (5).

6 $$\begin{aligned} \mathcal {L}_\textrm{empty} = \mathbb {E}_{\textbf{r} \sim \mathcal {D}} \left[ \int _{t_{n}}^{z(\textbf{r})-\epsilon } \left( w(t)\right) ^2 dt \right] \end{aligned}$$

One of the main strengths of our method during the initial phase of our progressive optimization is the supervision via an optical flow loss $\mathcal {L}_{f}$ in both temporal directions as described in Eq. (8). The estimated optical flow $\hat{F}_{k\rightarrow k\pm 1}$ is induced via finding the surface point for a given ray at time *k* in 3D with the help of the predicted depth $\hat{z}$ using the de-projection from 2D to 3D $\pi _{3D}$ and then transforming the point to the adjacent timestep $k\pm 1$ using the relative camera transformation between the adjacent camera views $\left[ R|T\right] _{k\rightarrow k\pm 1}$. The resulting 3D point we then project from 3D to 2D via $\pi _{2D}$ using the known camera intrinsics and compared to the initial pixel location $\textbf{p}(\textbf{r})$ at time k (see Eq. (7)). Note that both $\pi _{2D}$ and $\pi _{3D}$ are obtained from LocalRF [18].

7 $$\begin{aligned} \hat{\mathcal {F}}_{k\rightarrow k\pm 1}(\textbf{r})= &   \textbf{p}(\textbf{r}) - \pi _{2D} \left( \left[ R|T\right] _{k\rightarrow k\pm 1} \pi _{3D}(\textbf{r}, \hat{z}) \right) \end{aligned}$$

8 $$\begin{aligned} \mathcal {L}_{f}= &   \frac{1}{|\mathcal {R}|}\sum _{\textbf{r}\in \mathcal {R}} \left\| \hat{\mathcal {F}}_{k\rightarrow k\pm 1}(\textbf{r}) - \mathcal {F}_{k\rightarrow k\pm 1}(\textbf{r})\right\| _{1} \end{aligned}$$

All the aforementioned losses are aggregated into our final loss function $\mathcal {L}$ and we propose to balance their absolute values with $\lambda _{z,f}$. It is essential to highlight that we employ all three loss terms to optimize our method FLex. The camera poses, however, are only optimized by the optical flow loss $\mathcal {L}_{f}$ (see Fig. 1). Note that the optical flow loss $\mathcal {L}_{f}$ is entirely removed after 20% of the *refinement phase* of the local model, ensuring an early pose convergence and an increased focus on the reconstruction quality following it.

9 $$\begin{aligned} \mathcal {L} = \mathcal {L}_{rgb} + \lambda _z \mathcal {L}_{z} + \lambda _f \mathcal {L}_{f} \end{aligned}$$

## Experiments

### Dataset and evaluation metrics

We systematically assess the efficacy of our approach using the publicly available StereoMIS [22] dataset, recorded using a stereo endoscope of a da Vinci Xi robot; ground-truth camera trajectories are measured using the forward kinematics. In total we extract five sequences for general comparison, each 1000 frames long equivalent to approximately 29 s per clip. Two of these scenes exhibit concurrent breathing motion and camera movement, while another two showcase pronounced non-rigid deformations induced by surgical tools amidst less strong changes in camera perspective. The remaining scene presents a nearly static environment accompanied by rapid camera movements. Furthermore, we create two additional longer sequences to study the method’s behavior given a larger temporal and spatial extent. One sequence contains deformations induced by surgical tools and consists of 5000 frames, while the other scene incorporates more rapid camera motion and comprises 4000 frames. We will make our data and train/test splits public to allow for easier comparisons in the future. For quantitative evaluation, we follow [7] by holding out certain frames during training and evaluating them with PSNR, SSIM [26], and LPIPS [27] metrics (denoted with subscripts “a” and “v” for AlexNet and VGG backbones available in [27]) and L1 distance metric, indicated in mm, to assess the captured geometry by comparing the induced and stereo-estimated depth images. We consider the stereo-estimated depth as *ground truth* since a measured depth is not available. For evaluating camera pose accuracy, we follow the approach of robust-pose estimation [22] and make use of root-mean-squared absolute trajectory error (ATE-RMSE), relative translational and rotational pose errors (RPE-Trans and RPE-Rot).

Since the focus of our work lies in addressing the challenges of temporal and spatial scalability and joint camera pose estimation in endoscopic reconstruction we do not evaluate our method on the EndoNeRF Dataset [7], which features short sequences of 150 frames and a static camera position. We believe however that the data extracted from the StereoMIS [22] dataset presented in this work represents a more realistic scenario and run all relevant prior works as baselines on this data.

### Implementation details

We set the dimension of the spatial feature grids to 512 for (*x*, *y*, *z*), and the temporal dimension is set to half of the amount of represented images, following the dimensions in HexPlane [24]. Except for our method with pose optimization and LocalRF [18], we use robust-pose estimation [22] to estimate the camera poses. In the experiment tables, we use $\hbox {HexPlane}^{\dagger _1}$ to denote an improved version with scene contraction[28], depth loss, and optical flow loss; and $\hbox {LocalRF}^{\dagger _2}$ to depict a version of it which substitutes the monocular with the stereo depth estimation. We add these additional architecture modifications to optimize both methods for an endoscopic environment and make them more comparable to our proposed FLex.

We set $\lambda _{z} = 0.01$ and $\lambda _{f} = 1.0$ as illustrated in Eq. (9) and $t_{k}=100$ and $t_{d} = 1.0$. Overall, we train our method without pose optimization for 100 iterations per frame with a batch size of 4096 rays, which takes approximately 7 h on up to 40 GB of an Nvidia Tesla A100 and FLex with pose optimization for an additional 100 iterations per frame during the prior progressive optimization which yields approximately 20 h on the same hardware configuration. In comparison, HexPlane, for the exact same settings, takes approximately 6 h to train. Additionally, during inference, both our methods and HexPlane achieve approximately 0.3 FPS, which is slower compared to the Gaussian splatting baselines [13, 15]. It is important to note that inference and training speed are not the primary focus of this work and could be enhanced by incorporating Gaussian splatting approaches as backbone models in future developments. We do not mask the tools in any experiment since they are integral for the surgical procedure and should be present in any reconstruction. In addition, we use RAFT [23] for estimating both optical flow from frame-to-frame ${\mathcal {F}}_{k\rightarrow k\pm 1}(\textbf{r})$ for both directions and for obtaining stereo depth $z(\textbf{r})$, which we both use as pseudo-ground-truth for model optimization. We use the same pseudo-ground-truth depth for all models to ensure a fair comparison.

**Table 1:** View synthesis quality on StereoMIS dataset

Model	PSNR $\uparrow$	SSIM $\uparrow$	$\text {LPIPS}_a$ $\downarrow$	$\text {LPIPS}_v$ $\downarrow$	L1 Distance $\downarrow$
EndoNeRF [7]	21.99	0.590	0.496	0.514	–
EndoSurf [8]	25.18	0.622	0.528	0.529	8.105
ForPlane [10]	30.35	0.783	0.208	0.301	23.717
$\hbox {LocalRF}^{\dagger _2}$ [18]	27.41	0.781	0.245	0.288	4.576
$\hbox {HexPlane}^{\dagger _1}$ [24]	30.85	0.819	0.211	0.273	1.532
Endo4DGS [13]	28.01	0.734	0.265	0.350	–
Deform3DGS [15]	27.57	0.751	0.322	0.361	–
FLex w/o Pose Optim. (Ours)	**31**.**10**	**0**.**836**	0.200	**0**.**244**	1.456
FLex w/ Pose Optim. (Ours)	30.62	0.818	**0**.**179**	0.245	**1**.**273**

The metrics are computed as an average for five 1000-frame sequences. L1 Distance is computed between the synthesized and the ground-truth depth images in mm. The best result for each metric is marked in bold

### Quantitative and qualitative results

**Fig. 2:**
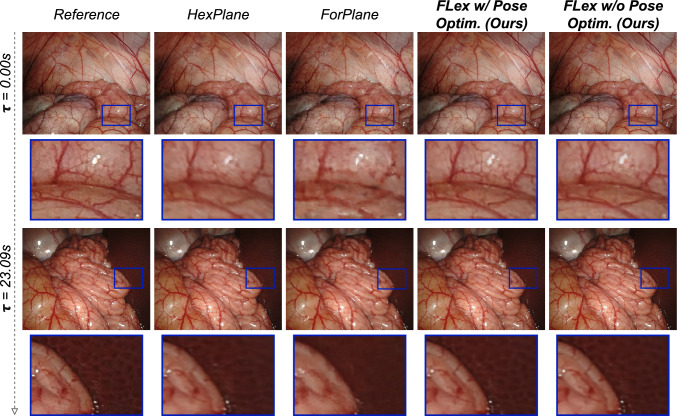
Qualitative results showing two images at two different timesteps from a 1000-frame scene with breathing deformations and camera motion. The reference image is the ground truth image, and the dark blue framed images are the zoomed-in sections of the upper images. Especially the zoomed-in sections highlight a finer image quality of both FLex variants compared to HexPlane [24] and ForPlane [10] (strongest two baselines)

**Fig. 3:**
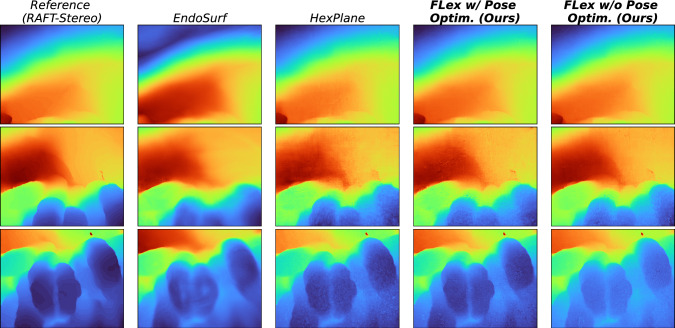
Qualitative results for depth prediction showing three images at different timesteps from a 1000-frame scene with breathing deformations and camera motion. The reference image is the pseudo-ground truth obtained from RAFT-Stereo

We conduct a comprehensive comparison of the proposed method, both with and without pose optimization, against the latest published state-of-the-art (SoTA) NeRF methods designed for endoscopy [7, 8, 10] and two additional baselines [18, 24] that are not specifically designed for endoscopy. The results in Table 1, summarizing the average results across all 5 scenes, demonstrate that FLex without pose optimization consistently outperforms all baselines and notably surpasses the current endoscopic SoTA, ForPlane, by 5.3 SSIM while achieving substantially better geometry reconstruction as measured by L1 distance. In addition FLex with pose optimization also manages to outperform ForPlane in all metrics and is competitive to FLex without pose optimization and the improved $\hbox {HexPlane}^{\dagger _1}$. These quantitative findings are substantiated by our qualitative results presented in Fig. 2, highlighting that FLex, with and without prior poses, renders images with clearer high-frequency details and less blur than the most competitive baselines, our improved $\hbox {Hexplane}^{\dagger _1}$ version and ForPlane. Similarly, we showcase in Fig. 3 that our method achieves a higher geometric reconstruction quality.

#### Comparison with Gaussian splatting-based methods

We also compare with recent endoscopic Gaussian splatting works [13, 15] and outperform them significantly, as shown in Tables 1 and 2. We could not reproduce the exact data splits that both Endo4DGS [13] and Deform3DGS [15] use, since they do not release any data or information about the exact frames that were extracted from the StereoMIS dataset for their experiments. Therefore the numbers presented here differ substantially, as Endo4DGS [13] reports results based on a single scene (approximately 200 frames) of StereoMIS, while Deform3DGS [15] reports results from three scenes (around 150 frames each). We use at least 1000 frames per scene. To ensure a fair comparison, we scale the number of training iterations according to the length of our scenes. Additionally, to enable easier comparisons in future, we have made all the data used in our experiments publicly available. As can be seen in Table 2, both GS-based methods perform worse whenever camera movement or tool interaction is introduced. This is due to the assumptions that both works make, where the first frame is used to initialize scene geometry. If a large scene movement is introduced either by camera motion or a tool the GS-based methods struggle to model the additional geometry. While all methods perform worse in scenes with extreme deformations, e.g., caused by rapid tool movement, NeRF methods tend to model it better.

**Table 2:** Per sequence comparison of novel synthesis quality on StereoMIS dataset

Model	Def	Cam. Motion	Tool	PSNR $\uparrow$	SSIM $\uparrow$	LPIPS_a $\downarrow$	LPIPS_v $\downarrow$	L1 Dist. $\downarrow$
EndoNeRF [7]	$\checkmark$	$\checkmark$		25.28	0.628	0.507	0.500	–
		$\checkmark$		11.22	0.546	0.517	0.573	–
	$\checkmark$		$\checkmark$	23.87	0.569	0.501	0.517	–
	$\checkmark$	$\checkmark$		26.56	0.636	0.409	0.456	–
	$\checkmark$		$\checkmark$	23.03	0.569	0.545	0.524	–
EndoSurf [8]	$\checkmark$	$\checkmark$		25.14	0.619	0.516	0.533	1.143
		$\checkmark$		29.81	0.766	0.513	0.552	1.895
	$\checkmark$		$\checkmark$	23.42	0.582	0.493	0.505	14.025
	$\checkmark$	$\checkmark$		25.39	0.609	0.482	0.505	17.147
	$\checkmark$		$\checkmark$	22.14	0.533	0.618	0.567	6.318
LerPlane [9]	$\checkmark$	$\checkmark$		29.47	0.766	0.182	0.292	8.175
		$\checkmark$		36.17	0.900	**0**.**139**	0.273	20.875
	$\checkmark$		$\checkmark$	27.28	0.710	0.272	0.355	30.702
	$\checkmark$	$\checkmark$		32.70	0.850	**0**.**149**	0.214	30.325
	$\checkmark$		$\checkmark$	26.11	0.690	0.296	0.373	28.511
$\hbox {LocalRF}^{\dagger _2}$ [18]	$\checkmark$	$\checkmark$		29.02	0.818	0.177	0.233	3.926
		$\checkmark$		35.07	0.895	0.172	**0**.**235**	2.701
	$\checkmark$		$\checkmark$	22.54	0.701	0.318	0.352	6.424
	$\checkmark$	$\checkmark$		31.22	0.841	0.166	0.209	4.418
	$\checkmark$		$\checkmark$	19.21	0.649	0.394	0.409	5.411
$\hbox {HexPlane}^{\dagger _1}$ [24]	$\checkmark$	$\checkmark$		31.50	0.854	0.170	0.233	1.098
		$\checkmark$		36.70	0.916	0.178	0.251	2.497
	$\checkmark$		$\checkmark$	27.13	0.745	0.258	0.317	**1**.**397**
	$\checkmark$	$\checkmark$		33.04	0.872	0.162	0.206	1.154
	$\checkmark$		$\checkmark$	25.90	0.710	0.287	0.359	**1**.**516**
Endo4DGS [13]	$\checkmark$	$\checkmark$		28.32	0.772	0.229	0.302	–
		$\checkmark$		31.83	0.832	0.218	0.359	–
	$\checkmark$		$\checkmark$	24.39	0.617	0.365	0.453	–
	$\checkmark$	$\checkmark$		31.72	0.832	0.161	0.219	–
	$\checkmark$		$\checkmark$	23.77	0.619	0.352	0.415	–
Deform3DGS [15]	$\checkmark$	$\checkmark$		27.11	0.734	0.322	0.362	–
		$\checkmark$		31.84	0.839	0.325	0.383	–
	$\checkmark$		$\checkmark$	25.75	0.743	0.305	0.338	–
	$\checkmark$	$\checkmark$		29.31	0.766	0.281	0.320	–
	$\checkmark$		$\checkmark$	23.85	0.674	0.377	0.401	–
Ours w/o Pose Optim.	$\checkmark$	$\checkmark$		**31**.**91**	**0**.**875**	0.155	0.201	1.234
		$\checkmark$		**37**.**03**	**0**.**917**	0.173	0.242	1.708
	$\checkmark$		$\checkmark$	27.21	0.773	0.242	0.274	1.533
	$\checkmark$	$\checkmark$		**33**.**71**	**0**.**889**	0.152	**0**.**184**	**1**.**052**
	$\checkmark$		$\checkmark$	25.65	0.724	0.279	0.318	1.752
Ours w/ Pose Optim.	$\checkmark$	$\checkmark$		31.53	0.851	**0**.**139**	**0**.**196**	**1**.**052**
		$\checkmark$		35.25	0.885	0.180	0.262	**1**.**093**
	$\checkmark$		$\checkmark$	**28**.**07**	**0**.**801**	**0**.**187**	**0**.**246**	1.453
	$\checkmark$	$\checkmark$		31.97	0.816	0.157	0.216	1.062
	$\checkmark$		$\checkmark$	**26**.**30**	**0**.**736**	**0**.**230**	**0**.**304**	1.703

L1 Distance is computed between the synthesized and the ground-truth depth images in mm. $\hbox {HexPlane}^{\dagger _1}$ stands for an optimized baseline with scene contraction, depth loss, and optical flow loss, while $\hbox {LocalRF}^{\dagger _2}$ is the vanilla LocalRF, but optimized via stereo depth. Numbers highlighted in **bold** indicate the best result and underlined the second best for each metric and each scene, respectively

### Ablation studies

**Table 3:** Ablation studies on loss functions and the use of local models compared to having one global representation ($\hbox {HexPlane}^{\dagger _1}$) on a 1000-frame StereoMIS scene

Model	PSNR $\uparrow$	SSIM $\uparrow$	$\text {LPIPS}_a$ $\downarrow$	$\text {LPIPS}_v$ $\downarrow$	L1 Dist. $\downarrow$
$\hbox {HexPlane}^{\dagger _1}$ [24] + $w/o \mathcal {L}_z$ + $w/o \mathcal {L}_f$	**31**.**98**	**0**.**864**	**0**.**158**	**0**.**218**	104.272
$\hbox {HexPlane}^{\dagger _1}$ [24] + $w/o \mathcal {L}_f$	31.63	0.857	0.164	0.228	1.142
$\hbox {HexPlane}^{\dagger _1}$ [24]	31.64	0.857	0.164	0.228	**1**.**117**
FLex w/o Pose Optim. (Ours) + $w/o \mathcal {L}_f$	32.25	**0**.**884**	**0**.**141**	**0**.**183**	0.975
FLex w/o Pose Optim. (Ours)	**32**.**26**	**0**.**884**	0.142	0.184	**0**.**953**

L1 distance is computed between the synthesized and the estimated stereo depth images in mm. The best results are marked in bold. Note that the global representation cannot jointly optimize for camera poses

**Table 4:** Ablation study on longer StereoMIS sequences

Model	Frame #	PSNR $\uparrow$	SSIM $\uparrow$	$\text {LPIPS}_a$ $\downarrow$	$\text {LPIPS}_v$ $\downarrow$	L1 Dist. $\downarrow$
$\hbox {HexPlane}^{\dagger _1}$ [24]	4000	24.79	0.614	0.545	0.510	4.856
FLex w/o Pose Optim. (Ours)	4000	**26**.**09**	**0**.**661**	**0**.**498**	**0**.**469**	**3**.**567**
$\hbox {HexPlane}^{\dagger _1}$ [24]	5000	28.55	0.718	0.453	0.471	1.902
FLex w/o Pose Optim. (Ours)	5000	**29**.**97**	**0**.**773**	**0**.**386**	**0**.**413**	**1**.**704**

L1 distance is computed between the synthesized and the estimated stereo depth images in mm. The best results are marked in bold

**Table 5:** Average pose accuracy on StereoMIS dataset

Model	ATE-RMSE $\downarrow$	RPE-Trans $\downarrow$	RPE-Rot $\downarrow$
Robust-pose estimation [22]	$\mathbf {2.164}\pm 2.68e-1$	$\mathbf {0.073}\pm 3e-5$	$\mathbf {0.043}\pm 2e-6$
$\hbox {LocalRF}^{\dagger _2}$ [18]	$7.704\pm 1.506$	$0.160\pm 8e-4$	$0.119\pm 2e-5$
FLex w/ Pose Optim. (Ours)	$2.565\pm 1.6e-1$	$0.127\pm 9e-4$	$0.102\pm 4e-6$

ATE-RMSE and RPE-Trans are in mm, RPE-Rot is in degrees. The best results are marked in bold

Table 3 displays the impact of the different loss functions to our method as well as justifies the usage of local 4D representations over one global representation as in HexPlane. Although relying only on the color loss $\mathcal {L}_{rgb}$ achieves slighly better visual results for HexPlane, but poor geometric reconstruction. Both the depth loss $\mathcal {L}_{z}$ and the optical flow $\mathcal {L}_{f}$ loss help both the global representation as well as the local models to achieve a more accurate scene geometry.

To showcase the proposed models efficacy in significantly longer recordings we also evaluate it on sequences of up to 5000 frames, representing recordings of around 2.25 min length. We compare to the best baseline, the improved version of $\hbox {HexPlane}^{\dagger _1}$. Ensuring a reasonable hardware memory cap of 16 GB vRAM, we set the spatial grid sizes to 128. Additionally, we set the maximum temporal dimensions for our method and HexPlane to 50 per local model and 100, respectively, and we train the models for 100 iterations per frame. As displayed in Table 4, our method preserves its reconstruction quality even for long recordings, achieving a gain of 1.42 db in PSNR and 5.5 SSIM over the baseline for the 5000 frame experiment. This substantial performance gain over $\hbox {HexPlane}^{\dagger _1}$ is achieved due to the local model architecture, which enables it to scale with the amount of frames as needed without running out of vRAM or network capacity.

### Pose accuracy

We compare FLex against a SoTA method in visual odometry for endoscopic scenes, robust-pose estimation [22], and the original LocalRF [18] on 3 sequences each with 1000 frames. As highlighted in Table 5, FLex performs competitively achieving close results to the fully supervised robust-pose estimation and outperforms LocalRF by a good margin since LocalRF cannot model the scene deformations. However, please note that this task is not the main focus of our work and can be improved using robust optimization and globally consistent methods in future.

## Conclusion

In this work, we present FLex, a novel method for reconstructing pose-free, long surgical videos with challenging tissue deformations and camera motion. Our approach successfully eliminates the reliance on prior poses by jointly optimizing for 4D reconstruction and camera trajectory via optical flow and depth supervision in a progressive manner. FLex improves upon the scalability of dynamic NeRFs for larger scenes thus becoming more applicable to boundlessly long surgical recordings, while improving over current methods on the StereoMIS dataset in terms of novel view synthesis with competitive pose accuracy. In our future work, we see great promise in combining local scene representations and optical flow supervision with dynamic Gaussian splatting methods to allow for faster pose-free reconstruction and real-time rendering. We believe that FLex can pave the way toward more easily accessible, realistic and reliable 4D endoscopy reconstructions to improve post-surgical analysis and medical education.

## Supplementary Information

Below is the link to the electronic supplementary material.

Supplementary file 1 (pdf 149 KB)
