# Supplementary material for: FLex: joint pose and dynamic radiance fields optimization for stereo endoscopic videos
Source: Int J Comput Assist Radiol Surg. 2025 Jul 21;21(1):137–46. doi: 10.1007/s11548-025-03446-6 (PMC12929345; doi:10.1007/s11548-025-03446-6)
Supplement: Supplementary file 1 — (pdf 149 KB) [file 11548_2025_3446_MOESM1_ESM.pdf]

FLex: Joint Pose and Dynamic Radiance Fields Optimization for Stereo Endoscopic Videos

Florian Stilz[1*†], Mert Karaoglu[1,2†], Felix Tristram[1†], Nassir Navab[1], Benjamin Busam[1], Alexander Ladikos[2]

[1]Chair for Computer Aided Medical Procedures, Technical University of Munich, Boltzmannstr. 3, Garching, 85748, Germany.
[2]Imfusion GmbH, Agnes-Pockels-Bogen 1, München, 80992, Germany.

*Corresponding author(s). E-mail(s): florian.stilz@tum.de;
†These authors contributed equally to this work.

Keywords: 3D Reconstruction, Neural Rendering, Robotic Surgery, Pose Optimization

1 Additional Implementation Details

In our local models, we set the dimension of the spatial feature grids to 512 for (x, y, z), and the temporal dimension is set to half of the amount of represented images, following the dimensions in HexPlane [1]. The feature dimension is 72 in total for both density and color. For a fair comparison, we ensure equal capacity for all methods using explicit data structures [1–3], meaning all those methods have equal feature grid dimensions spatially and proportionally to the covered image sequence for the temporal dimension (see numbers above). An equal capacity is important as bigger feature grids can naturally achieve better results and our comparisons should be as agnostic as possible to that hyperparameter. Additionally, we adopt a coarse-to-fine approach as in HexPlane [1] to start with a lower grid resolution and increase over time to the settings mentioned above.

2 Additional Results

This section provides more detailed result information.

Table 1 Per sequence comparison of pose accuracy on StereoMIS dataset. LocalRF†[2] is the vanilla LocalRF, but optimized via stereo depth. The ATE-RMSE and the RPE-Trans are in mm, and the RPE-Rot is in degrees.

Model	Def.	Cam. Motion	Tool	ATE-RMSE ↓	RPE-Trans ↓	RPE-Rot ↓
Robust	✓	✓		2.407	**0.068**	**0.043**
Pose		✓		2.640	**0.080**	**0.054**
Estimation	✓	✓		**1.444**	**0.071**	**0.032**
	Average			$\mathbf{2.164} \pm 2.68e-1$	$\mathbf{0.073} \pm 3e-5$	$\mathbf{0.043} \pm 2e-6$
	✓	✓		8.210	0.155	0.136
LocalRF†[2]		✓		8.888	0.198	0.143
	✓	✓		6.013	0.128	0.079
	Average			7.704 ± 1.506	$0.436 \pm 8e-4$	$0.119 \pm 2e-5$
Ours w/	✓	✓		**2.106**	0.099	0.090
Pose		✓		**2.509**	0.113	0.123
Optim.	✓	✓		3.081	0.168	0.093
	Average			$2.565 \pm 1.6e-1$	$0.127 \pm 9e-4$	$0.102 \pm 4e-6$

References

[1] Cao, A., Johnson, J.: Hexplane: A fast representation for dynamic scenes. In: Proceedings of the IEEE/CVF Conference on Computer Vision and Pattern Recognition, pp. 130–141 (2023)

[2] Meuleman, A., Liu, Y.-L., Gao, C., Huang, J.-B., Kim, C., Kim, M.H., Kopf, J.: Progressively optimized local radiance fields for robust view synthesis. In: Proceedings of the IEEE/CVF Conference on Computer Vision and Pattern Recognition, pp. 16539–16548 (2023)

[3] Yang, C., Wang, K., Wang, Y., Dou, Q., Yang, X., Shen, W.: Efficient deformable tissue reconstruction via orthogonal neural plane. arXiv preprint arXiv:2312.15253 (2023)
